# The fitness of African malaria vectors in the presence and limitation of host behaviour

**DOI:** 10.1186/1475-2875-11-425

**Published:** 2012-12-19

**Authors:** Issa N Lyimo, Daniel T Haydon, Kasian F Mbina, Ally A Daraja, Edgar M Mbehela, Richard Reeve, Heather M Ferguson

**Affiliations:** 1 Environmental Sciences Thematic Group, Ifakara Health Institute, PO.BOX 53, Ifakara, Tanzania; 2 Boyd Orr Centre for Population and Ecosystem Health, College of Medicine, Veterinary and Life Sciences, University of Glasgow, Glasgow, G12, 8QQ, UK

**Keywords:** Malaria vectors, Fitness, Feeding success, Host species range, Host behaviour, Natural selection

## Abstract

**Background:**

Host responses are important sources of selection upon the host species range of ectoparasites and phytophagous insects. However little is known about the role of host responses in defining the host species range of malaria vectors. This study aimed to estimate the relative importance of host behaviour to the feeding success and fitness of African malaria vectors, and assess its ability to predict their known host species preferences in nature.

**Methods:**

Paired evaluations of the feeding success and fitness of African vectors *Anopheles arabiensis* and *Anopheles gambiae sensu stricto* in the presence and limitation of host behaviour were conducted in a semi-field system (SFS) at Ifakara Health Institute, Tanzania. In one set of trials, mosquitoes were released within the SFS and allowed to forage overnight on a host that was free to exhibit a natural behaviour in response to insect biting. In the other, mosquitoes were allowed to feed directly on from the skin surface of immobile hosts. The feeding success and subsequent fitness of vectors under these conditions were investigated on six host types (humans, calves, chickens, cows, dogs and goats) to assess whether physical movements of preferred host species (cattle for *An. arabiensis*, humans for *An. gambiae s.*s.) were less effective at preventing mosquito bites than those of common alternatives.

**Results:**

*Anopheles arabiensis* generally had greater feeding success when applied directly to host skin than when foraging on unrestricted hosts (in five of six host species). However, *An. gambiae s.s.* obtained blood meals from free and restrained hosts with similar success from most host types (four out of six). Overall, the blood meal size, oviposition rate, fecundity and post-feeding survival of mosquito vectors were significantly higher after feeding on hosts free to exhibit behaviour, than those who were immobilized during feeding trials.

**Conclusions:**

Allowing hosts to move freely during exposure to mosquitoes was associated with moderate reductions in mosquito feeding success, but no detrimental impact to the subsequent fitness of mosquitoes that were able to feed upon them. This suggests that physical defensive behaviours exhibited by common host species including humans do not impose substantial fitness costs on African malaria vectors.

## Background

Many plants and animals actively defend themselves from ectoparasites by chemical and/or behavioural responses [1-3]. The role of host defensive mechanisms (e.g. such as the secretion of chemical compounds) in driving the host specificity of agricultural pest insects has been well documented [1,4,5]. In contrast, relatively little is known about the importance of physical behaviours mounted by vertebrate hosts in generating selection for host specificity in insect disease vectors [6]. Of all the insect vectors of human disease, *Anopheles* mosquitoes are responsible for the greatest loss of life and morbidity through their role in malaria transmission [7,8].

The frequency with which mosquito vectors feed on humans, and adult mosquito survival, are key determinants of malaria transmission intensity [7,9]. Both of these phenomena may be influenced by host physical movements. Specifically hosts that are free to exhibit behavioural responses can prevent mosquitoes from biting [10,11], and/or interrupt their blood feeding [12]. These host responses could limit parasite transmission by reducing host – vector contact rates and increasing the risk of mortality in host-seeking mosquitoes [13]. Alternatively, host physical movements that do not kill mosquitoes but divert them to other hosts could enhance parasite transmission by increasing the number of hosts that vectors contact during their lifetime [12,14]. Consequently, host behavioural responses could have substantial impacts on the fitness of both malaria parasites and their vectors, and correspondingly generate selection for specificity on poorly defensive host types.

Studies of mosquitoes and other haematophagous insects have shown that their feeding success on vertebrates can be significantly reduced by host defensive behaviours [12,15,16]. The effectiveness of host behavioural responses has been shown to vary between host species [17], individuals [18], and also in association with additional factors such as whether hosts are infected by parasites [15], and the overall density of biting insects [19,20]. The consequences of such host behaviours may be non-linearly related to ectoparasite fitness. For example the feeding and reproductive success of fleas is significantly reduced by strong host behavioural and immune defenses [21-23], but moderate host defensive behaviours can enhance their blood intake and survival [23]. Consequently, the net impact of host behaviours on the feeding success of vectors may be context-specific, and ideally should be assessed against the background of host physical movements that they are most likely to encounter when foraging within natural settings.

The strong preference of African malaria vectors such as *Anopheles gambiae s*.s for human-feeding has been speculated to be the result of the poor anti-mosquito defensive behaviours of people relative to other animal alternatives. A reason cited for why humans have been assumed to be weakly defensive hosts is that they are typically asleep during the night-time hours where malaria vector biting activity is concentrated. However, other host species which malaria vectors could feed upon (e.g. livestock, dogs) also sleep during these hours so this behaviour does not uniquely distinguish humans. While the associations between host-specific defensive behaviours and insect host preferences have been experimentally investigated in Diptera foraging on birds [15], rodents [24] and livestock [25], testing this hypothesis on human malaria vectors has been restricted by ethical constraints arising from the requirement to monitor human behaviour in response to attack by potentially infected mosquito vectors. To overcome this obstacle, here experiments were conducted in an experimental semi-field-system (SFS) situated at the Ifakara Health Institute (IHI) in Tanzania in which vector – human interactions can be studied under relatively natural conditions using only mosquitoes that are guaranteed to be malaria-free.

Within this setting, the feeding success and subsequent fitness of the two most important African malaria vectors *Anopheles arabiensis* and *An. gambiae s.s* were compared under conditions when hosts were free to exhibit natural physical behaviours in response to mosquitoes, and when mosquitoes were directly applied to the skin of immobile hosts. The assumption was that most forms of host defensive behaviour towards mosquito biting are eliminated when mosquitoes are applied directly to the skin surface of immobile hosts. It is, however, possible that some subtle host behaviours, such as skin rippling, can still occur even under these conditions. Thus, the experimental design employed here allowed comparison of mosquito feeding and fitness in response to host behavioural limitation, but not necessarily its complete absence. The following hypotheses were tested: (1) mosquito feeding success and subsequent fitness is greater when host behaviour is restricted, and (2) the relative efficiency of host physical behaviours in preventing mosquito biting is correlated with the documented host preferences of these mosquito vectors (e.g. cattle for *An. arabiensis*[26], and humans for *An. gambiae* s.s [27]). In testing the latter hypothesis, the relative efficiency of host behaviour was estimated by the magnitude of difference in mosquito feeding success upon ‘free’ and behaviourally-restricted hosts. A specific prediction was that the host species which are least effective at repelling mosquito biting would be cows for *An. arabiensis,* and humans for *An. gambiae* s.s. By characterizing the fitness costs imposed by host physical movement, this study can shed light on the potential for malaria vectors to adapt to new host species in response to the mass coverage of public-health interventions that specifically protect humans.

## Methods

### Study site and mosquito colonies

The study was conducted at the Ifakara Health Institute (IHI) in the Kilombero valley, Tanzania. Here high levels of malaria transmission are sustained year-round by *An. arabiensis, An. gambiae s.s*, and *Anopheles funestus*. Experiments were conducted using *An. arabiensis* and *An. gambiae* s.s from colonies maintained at the IHI. The *An. arabiensis* colony was established a few months before the start of experiments with individuals from Sagamaganga village (~15 km from IHI) and is maintained in a semi-field insectary (25 – 32°C, 51 – 90% R.H [28]). The *An. gambiae* s.s colony was established with individuals from Njage village in 1996 (~70 km from IHI) and is maintained in an indoor insectary (26 ± 2.5°C 80 ±10% R. H). Both colonies are maintained on human-blood provided thrice weekly by arm feeding. Female mosquitoes used in these experiments were provided with a 10% glucose solution *ad libitum* from eclosion, but deprived of this for 6 hours prior to host exposure in these experiments. All mosquitoes used in these experiments were adult females that had not been blood fed prior to experimentation, and were 4–6 days at the time of exposure to hosts.

### Mosquito feeding assays

A series of trials were conducted in which the feeding success (probability of obtaining a blood meal, blood meal size) and subsequent fitness (fecundity and survival) of cohorts of *An. arabiensis* and *An. gambiae s.s* were estimated after they were released inside a chamber (9.1 × 9.6 × 3.7 m) of a netting – enclosed SFS that contained a vertebrate host situated within an experimental hut (3.5 × 4 × 2.5 m, [29]). On each night of experiments, an individual host that was either a human, cow, dog, goat, or chicken was randomly allocated for placement in the hut [30]. Two different age groups of cattle were tested: adult cows and calves. Within other host types, animals were roughly of the same age and size. Mosquitoes were released into the chamber for a twelve hour period between 7 pm – 7 am which is coincident with their natural daily host seeking period [31]. During this period mosquitoes had opportunity to attempt to feed on a host within the SFS, that was free to mount physical behaviours against mosquito bites. The following morning, mosquitoes were recaptured and their feeding success recorded through visual inspection (either unfed or blood fed). All mosquitoes recaptured alive and blood fed were retained for fitness measurements as described below. Six replicates of each host and vector species combination were conducted, with a different host individual being used in each replicate of a given host species treatment (for each mosquito species: 200 mosquitoes per host individual x 6 host individuals per host treatment = 1,200 mosquitoes per host treatment).

Additional trials were conducted using the same host individuals that participated in the semi-field experiments, but under conditions where hosts were unable to mount any physical movements during mosquito feeding. During these trials, a transparent plastic holding cup containing 10 unfed female *An. arabiensis* or *An. gambiae s.s* females was directly applied to the skin surface of a host for a period of 15 minutes during the day (9 am -5 pm). Human hosts who were volunteers from the research team were asked to apply the mosquito holding cup directly to the skin of their forearm and refrain from moving until the trial was complete. Calves, cows and goats were prevented from moving by placing them within a metal livestock holding stall while cups containing mosquitoes were applied to their flank, neck, thigh or ears. Dogs and chickens were held by their owners with mosquito cups applied to their flank. As with semi-field experiments, trials using six different host individuals from each host type were conducted. Further replication was conducted at the level of host individual by using three mosquito holding cups per host individual to yield a sample size of 180 mosquitoes of each vector species in each host treatment (10 mosquitoes/cup × 3 cups/host individual × 6 host individual/host type = 180 mosquitoes per host type). For *An. arabiensis,* all experiments with restrained hosts were conducted on the day following the semi-field experiments. For *An. gambiae* s.s., two out of the six experimental replicates of all host types were also conducted on the day following semi-field experiments, with the remainder being conducting ~5 months after semi-field studies due to constraints in obtaining sufficient numbers of mosquitoes from the colony to perform both types of experiments simultaneously.

### Fitness measurements

All mosquitoes that succeeded in obtaining a blood meal were transferred into individual holding tubes (2.3 cm diameter × 9 cm depth) and held within a semi-field insectary for subsequent fitness measurements. Mosquito blood meal size was estimated indirectly on the basis of the amount of haematin excreted within 3 days after feeding. Individual mosquitoes were allowed to excrete haematin in 30 ml plastic tubes. The excreted haematin was dissolved in 1 ml of 1% lithium carbonate solution and its absorbance at 387 nm read in a spectrophotometer following an established protocol [32]. Haematin excretion does not begin the end of the blood digestion period [32]. Full digestion of blood meals requires > 12 hours in *Anopheles*, with blood imbibed by several Anopheles species including *An. gambiae* still being readily visible in their abdomen at 12 hours after feeding [33]. Consequently both mosquitoes used in semi-field experiments and host restraint experiments were moved into collection tubes prior to the expected onset of haematin excretion. Mosquitoes were subsequently moved into individual paper cups lined with damp filter paper to stimulate oviposition. Oviposition cups were inspected daily and the number of eggs laid within them counted. Mosquitoes remained in holding cups and were monitored daily until death to estimate their post-feeding survival. The number of eggs laid by mosquitoes was calculated by counting the egg batch under a dissecting microscope. Mosquitoes continued to be individually checked on a daily basis to measure their post-feeding survival which was estimated as the number of days from blood feeding until death.

### Ethical considerations

Mosquitoes used in these experiments had not been blood fed prior to use and were thus guaranteed to be free from malaria and other blood borne pathogens when released in the presence of hosts. All human volunteers were from the research team and provided written informed consent before participation. Rapid Diagnostic Tests were used to screen all human volunteers for malaria immediately before their planned participation in experiments to ensure they were not infected. Most animals used in these trials were volunteered for participation by their owners in the local community, with the exception of chickens that were purchased for this research and kept in a coop at the IHI. Prior written informed consent was obtained from all livestock keepers who provided animals. Only animals that had not been treated with any topical insecticide within 2 – 3 months prior to the proposed experiment were used. The Institutional Ethical Review Board (IRB) of the IHI (IHRDC/IRB/No.A015), the Tanzanian National Institute for Medical Research (NIMR1HQ/R.8a/Vol.IX/708) and the University of Glasgow granted ethical approval for this study.

### Statistical analyses

Statistical analysis was conducted to assess the impact of host behavioural limitation on the following mosquito fitness parameters: probability of obtaining a blood meal, blood meal size, oviposition rate (probability of laying eggs), fecundity (number of eggs laid) and post-feeding survival. In trials where *An. gambiae s.s* were directly applied to the skin of chickens and goats, few mosquitoes fed and none laid eggs. Consequently these two host species could not be included in further analysis of fitness traits in *An. gambiae s.s*.

Two of the outcome variables estimated in these experiments were binomial: the probability of blood feeding and of producing eggs. The remaining variables of mosquito blood meal size, fecundity and the number of days mosquitoes survived were continuous. Associations between these outcome variables and host species and behavioural conditions were analysed using generalized linear mixed effect models with appropriate link functions in the R statistical software [34]. For each vector species, the impact of host species, feeding condition (semi-field or applied directly to host skin) and their interaction on all mosquito traits were investigated. Within host species, the unit of replication was host individual (six per host species) which was fit as a random effect. The significance of these explanatory variables were tested by sequentially deleting them from a maximal model that contained all main effects, their interaction term and the random effect of host individual (as assessed by Likelihood Ratio Tests) [34]. When the interaction term was significant, the main effect of ‘feeding condition’ was analysed separately for each host species. When testing the significance of ‘feeding condition’ across different host types, the Holm-Bonferroni correction for multiple comparisons was applied [35]. All *P* and *Z*-values presented are after correction for multiple comparisons across host types.

Mosquito survival was analysed using the Cox Proportion Hazard Model (coxph) in the R statistical software [34]. A frailty function was used to incorporate the random effect of host individual into the Cox model while evaluating for the additional impact of host species, feeding condition, and their interaction. Initially, all three factors including the main effects and their interaction were fitted in the same statistical model. When the interaction term was significant, the main effect of feeding condition was analysed separately for each host species. All *P*-values presented for tests of feeding condition on the 6 different host types have been adjusted for multiple comparisons using the Holm-Bonferroni correction [35].

## Results

Overall, the recapture rates of mosquitoes exposed to hosts under SFS conditions were higher for *An. gambiae* s.s than *An. arabiensis*, but did not vary between host species for either mosquito species (*An. arabiensis*: χ^2^_5_ = 10.00, P = 0.07, and *An. gambiae* s.s: χ^2^_5_ = 7.87, P = 0.16, Table 1). The fitness traits of these mosquitoes when exposed to unrestrained and those of mosquitoes feeding on behaviourally-restricted hosts are as detailed below.

**Table 1:** **Recapture rate of *An. arabiensis *and *An. gambiae *s.s after being released to blood feed on free hosts under semi-field conditions (estimated from 6 replicates of each host-vector combination)**

**Recapture rates of vector species in semi-field conditions after exposure to free hosts**		
**Host species**	***An. arabiensis***	***An. gambiae s.s***
Calf	0.59 (0.45 – 0.71)	0.75 (0.55 – 0.91)
Chicken	0.46 (0.32 – 0.60)	0.53 (0.29 – 0.75)
Cow	0.56 (0.42 – 0.70)	0.65 (0.43 – 0.85)
Dog	0.32 (0.20 – 0.44)	0.87 (0.75 – 0.95)
Goat	0.41 (0.29 – 0.55)	0.72 (0.52 – 0.88)
Human	0.40(0.28 – 0.54)	0.82(0.66 – 0.94)

The numbers in brackets are 95% confidence intervals.

### Mosquito feeding success

**Figure 1:**
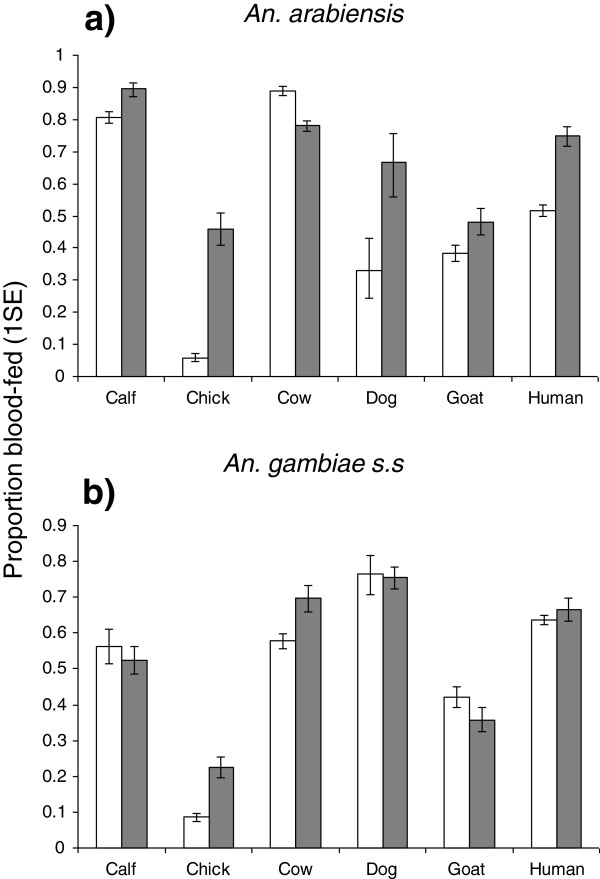
**Estimated proportion (±1 s.e) of *An. arabiensis *and *An. gambiae s.s *that succeeded in obtaining a blood meal after exposure to unrestrained hosts within a semi-field system (open box) and when applied directly to the skin surface of the same host individuals when they were immobile (grey box).** Six different individuals of each host type were used in each feeding condition.

The feeding success of *An. arabiensis* was related to host species in semi-field experiments with free moving hosts (χ^2^_5_ = 43.27, P < 0.001, Figure 1a), and in conditions where hosts were immobilized during mosquito exposure (χ^2^_5_ = 19.57, P = 0.001, Figure 1a). However the pattern of host-specific feeding success varied between these two experimental conditions; being higher on cows and calves than on all other host types under semi-field conditions (P < 0.05, in all cases, Figure 1a). However, *An. arabiensis* feeding success was similar on cows, calves, humans and dogs when hosts were restrained (P > 0.05, in all pairwise comparisons between cows/calves and other host types), but significantly lower on goats and chickens than on humans, calves and cows ( P < 0.05, in all pairwise comparions, Figure 1a). The feeding success of *An. gambiae* s.s was also host species-dependent under both experimental conditions (semi-field experiments: χ^2^_5_ = 20.29, P = 0.001; host restraint experiments: χ^2^_5_ = 22.77, P < 0.001, Figure 1b). In semi-field experiments, *An. gambiae s.s*. fed on humans to the same degree as all other host types with the exception of chickens, on which their feeding success was significantly reduced (Z = −3.73, P < 0.001, Figure 1b). Similarly in host restraint experiments, the feeding success of *An. gambiae* s.s on humans relative to other host types was only significantly reduced on chickens (Z = −3.99, P < 0.001, Figure 1b)*.*

For each vector species, the impact of host behavioural limitation on their feeding success varied between host species (host species × feeding condition: *An. arabiensis*, χ^2^_5_ = 126.94, P < 0.001, Figure 1a; *An. gambiae s.s*, χ^2^_5_ = 31.25, P < 0.001, Figure 1b). In *An. arabiensis*, the probability of obtaining a blood meal was significantly higher on most host species when their behaviour was limited (calves: χ^2^_1_ = 9.49, P =0.002, chickens: χ^2^_1_ = 140.46, P < 0.001, dogs: χ^2^_1_ = 24.92, P < 0.001, goats: χ^2^_1_ = 4.36, P = 0.04, and humans: χ^2^_1_ = 27.66, P < 0.001, Figure 1a). However, their feeding success on adult cows was significantly higher when these hosts were free to exhibit behaviours (χ^2^_1_ = 13.39, P < 0.001, Figure 1a). In contrast, the feeding success of *An. gambiae s.s* was relatively similar when hosts were free and behaviourally-restricted in many species (dogs, goats, calves and humans, P > 0.05 in all cases, Figure 1b). However, *An. gambiae* s.s. had a greater chance of obtaining a blood meal from chickens (χ^2^_1_ = 94.26, P < 0.001) and cows (χ^2^_1_ = 7.82, P = 0.03) when their behaviour was restricted (Figure 1b).

**Figure 2:**
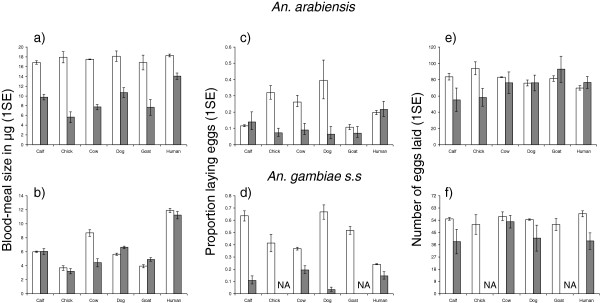
**Mean blood meal size, oviposition rate, and number of eggs produced by *An. arabiensis*, and *An. gambiae s.s *after blood feeding on different host types either under semi-field conditions where hosts were free to move (open box), or when applied directly to the skin surface of immobile hosts (grey box).** Error bars represents 1 standard error.

Mosquito blood meal size was also significantly influenced by the interaction between host species and feeding condition in both vector species (host species × feeding condition: *An. arabiensis*, χ^2^_5_ = 38.34, P < 0.001, Figure 2a, *An. gambiae s.s*, χ^2^_5_ = 73.15, P < 0.001, Figure 2b). When *An. arabiensis* were allowed to host seek under semi-field conditions, their blood meal size was similar across host types (χ^2^_5_ = 0.48, P = 0.99, Figure 2a). However in experiments where hosts were behaviourally limited, *An. arabiensis* obtained larger blood meals from humans than most other host types (P < 0.05 in all cases) except for dogs (Z = −2.34, P = 0.08). *Anopheles gambiae* s.s generally obtained larger blood meals from humans than from other host types both when foraging under semi-field conditions (P < 0.05 in all human-animal pairwise comparisons except for cows: Z = −1.79, P = 0.26), and when directly applied to the surface of host skin (P < 0.001 for all pairwise human-animal comparisons, Figure 2b).

*Anopheles arabiensis* obtained significantly larger blood meals from foraging on freely-moving hosts under semi-field conditions than when they were applied directly to the skin surface of immobile hosts (P < 0.001 for all host types), although the magnitude of difference due to feeding conditions varied between host species (Figure 2a). In contrast, the impact of limiting host behaviour on *An. gambiae s.s*. blood meal size was more variable. This vector acquired similarly-sized blood meals from foraging upon free or behaviourally restricted chickens, calves, goats, and humans (P > 0.05 in all cases, Figure 2b). However *An. gambiae* s.s obtained larger blood meals from freely moving than behaviourally-restricted cows (χ^2^_1_ = 72.93, P = 0.0006, Figure 2b), and smaller blood meals from dogs that were free to move than those who were behaviourally restricted (χ^2^_1_ = 6.83, P = 0.03, Figure 2b).

### Mosquito reproductive success

For mosquitoes that successfully acquired a blood meal, their probability of laying eggs depended on the interaction between host species and feeding condition (host species × feeding condition: *An. arabiensis*, χ^2^_5_ = 18.37, P = 0.002, Figure 2c; *An. gambiae s.s*, χ^2^_5_ = 87.48, P < 0.001, Figure 2d). *Anopheles arabiensis* had a higher probability of ovipositing after feeding on free rather than behaviourally-restricted chickens (χ^2^_1_ = 7.51, P = 0.02, Figure 2c), dogs (χ^2^_1_ = 20.60, P < 0.001, Figure 2c), and cows (χ^2^_1_ = 10.46, P < 0.01, Figure 2c). However, the oviposition success of *An. arabiensis* was similar under conditions where hosts were freely moving and behaviourally restricted for goats, calves, and humans, P > 0.05, Figure 2c). For all host types in which data were available (excludes chickens and goats), the oviposition rate of *An. gambiae s.s* was greater after feeding on freely moving than behaviourally restricted hosts (P < 0.05 in all cases, Figure 2d).

Restricting analysis to mosquitoes that laid at least one egg, the fecundity of blood fed *An. arabiensis* was unrelated to host species both when feeding under semi-field conditions (χ^2^_5_ = 5.83, P = 0.32, Figure 2e) and when directly applied to host skin (χ^2^_5_ = 2.44, P = 0.79, Figure 2e). Similarly, the fecundity of *An. gambiae* s.s. was unrelated to host species both when fed under semi-field conditions (χ^2^_5_ = 1.07, P = 0.96, Figure 2f), and when directly applied to host skin (χ^2^_3_ = 0, P > 0.99, Figure 2f). The fecundity of *An. arabiensis* was also unrelated to host behaviour conditions, with the average number of eggs produced by mosquitoes that fed from freely moving and behaviourally limited hosts being similar for all host types (P > 0.05 in all cases, Figure 2e). No data were available on fecundity of *An. gambiae s.s* after feeding on chickens and goats under conditions of behavioural limitation. However for most other host types, these mosquitoes had similar fecundity after feeding on hosts that were free to move under semi-field conditions, or immobile during blood feeding, (P > 0.05,, Figure 2f) with the exception of humans. In this case, *An. gambiae* s.s. fecundity was greater after feeding on humans under semi-field conditions than when directly applied to their skin surface (χ^2^_1_ = 8.96, P = 0.01, Figure 2f).

### Impact on the survival of mosquitoes

**Figure 3:**
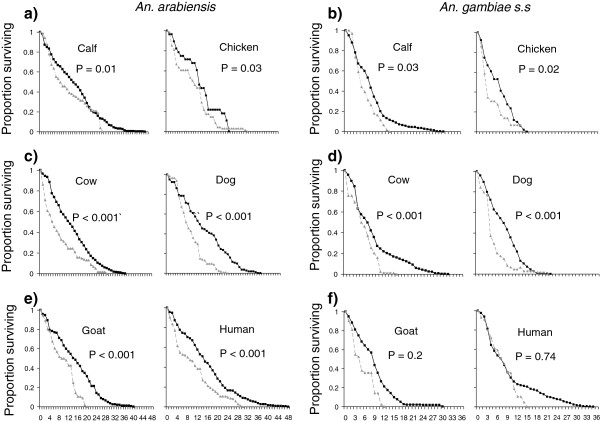
**Survival of *An. arabiensis *and *An. gambiae *s.s after feeding on different host types under semi-field conditions where hosts were free to move (black line), or when applied directly to the skin surface of immobile hosts (grey line).**

The post-feeding survival of *An. arabiensis* was similar on all host species when hosts were free to exhibit behaviours (χ^2^_5_ = 9, P = 0.1, Figure 3a-f). However, the post feeding survival of *An. arabiensis* was related to host species during trials where hosts were immobile during blood feeding (χ^2^_5_ = 20.2, P = 0.001, Figure 3a- f). Under these conditions, *An. arabiensis* survival was similar after feeding on humans relative to cows, calves, and chickens; but significantly higher after feeding on humans in comparison to dogs (χ^2^_1_ = 5.47, P = 0.02, Figure 3a-f), and goats (χ^2^_1_ = 10.74, P = 0.001, Figure 3a-f). The post-feeding survival of *An. arabiensis* was independent of their blood meal size in semi-field experiments (χ^2^_1_ = 1.10, P = 0.29), but increased with blood meal size in experiments with behaviourally-restricted hosts (χ^2^_1_ = 26.60, P < 0.001). The post-feeding survival of *An. gambiae* s.s was associated with host species both in trials where hosts were free (χ^2^_5_ = 27.0, P < 0.001, Figure 3a-f), and restricted from exhibiting physical behaviours (χ^2^_5_ = 22.3, P < 0.001, Figure 3a-f). When hosts were free to exhibit behaviours, the post-feeding survival of *An. gambiae* s.s on humans was significantly greater than on all other host species (P < 0.001 in all cases) except for cows on which it was similar (χ^2^_1_ = 1.16, P = 0.28, Figure 3a-f). When *An. gambiae* s.s. fed on behaviourally-restricted hosts, their post-feeding survival on humans was significantly greater than on all other host types (P < 0.001 in all cases). The post-feeding survival of *An. gambiae* s.s was positively associated with their blood meal size both in semi-field experiments (χ^2^_1_ = 46.00, P < 0.001), and in host behavioural restriction experiments (χ^2^_1_ = 22.90, P < 0.001).

The impact of host behavioural limitation on *An. arabiensis* survival did not depend on host species (host species × feeding condition: χ^2^_5_ = 6.00, P = 0.30, Figure 3a-f, Table 2) but was host-specific for *An. gambiae* s.s (host species × feeding condition: χ^2^_5_ = 18.00, P = 0.003, Figure 3a-f, Table 2). The post-feeding survival of *An. arabiensis* was greater after feeding on free hosts under semi-field experiments, than on behaviourally-restricted hosts of all host types (P < 0.05 in all cases, Figure 3a-f, Table 2). Similarly the post-feeding survival of *An. gambiae* s.s was greater after feeding on free rather than behaviorally –restricted calves (χ^2^_1_ = 6.85, P = 0.03, Figure 3a), chickens (χ^2^_1_ = 7.94, P = 0.02, Figure 3b), cows (χ^2^_1_ = 19.2, P = 0.0006, Figure 3c), and dogs (χ^2^_1_ = 45.5, P = 0.0006, Figure 3d), but similar under both feeding conditions for goats (χ^2^_1_ = 2.66, P = 0.2, Figure 3e) and humans (χ^2^_1_ = 0.11, P = 0.74, Figure 3f).

**Table 2:** **Estimated odds of mortality in *An. arabiensis *and *An. gambiae *s.s after blood feeding either under semi-field conditions where hosts were free to move during mosquito exposure, or when applied directly to the skin surface of immobile hosts**

**Odds of mortality (OR) after feeding on behaviourally-limited relative to unrestricted hosts**		
**Host species**	***An. arabiensis***	***An. gambiae s.s***
Calf	0.80 (0.61 – 1.04)	0.68 (0.51 – 0.91)
Chicken	0.54 (0.30 – 0.96)	0.43 (0.24 – 0.78)
Cow	0.56 (0.45 – 0.69)	0.55(0.42 – 0.72)
Dog	0.42 (0.29 – 0.60)	0.47 (0.38 – 0.58)
Goat	0.41 (0.29 – 0.58)	0.74 (0.51 – 1.06)
Human	0.58(0.45 – 0.74)	0.96(0.74 – 1.24)

The numbers in brackets are 95% confidence intervals.

## Discussion

This study demonstrates the potential impact of host physical movements on the feeding success and subsequent fitness of the African malaria vectors *An. arabiensis* and *An. gambiae s.s*. Consistent with initial prediction, there was some evidence that allowing hosts to make physical movements in the presence of mosquito biting did limit *Anopheles* feeding success. With the exception of cow hosts, blood feeding success rates in *An. arabiensis* applied directly to the skin of immobile hosts were generally higher than when they attempted to feed on the same individuals under more natural semi-field conditions. However this was not true for *An. gambiae* s.s. where host feeding rates were generally similar under semi-field conditions where hosts were free to move as when they were behaviourally-restricted (for four out of six host types). This suggests that host physical defensive behaviours may have a differential impact on these two vectors. While limiting host movement did consistently increase the feeding success of at least one vector species (*An. arabiensis*), it did not improve the quality of blood meals obtained by those that succeeded in feeding in terms of blood meal size, reproduction and survival. In fact, the oviposition and survival of mosquitoes that foraged on hosts under more natural semi-field conditions was generally greater than in those that had fed on behaviourally restricted hosts. These results indicate that while host physical movements may have moderate, host-specific impacts on malaria vector feeding probability, they do not diminish the quality of blood meals of the mosquitoes who are able to feed from them.

A key prediction that this study aimed to test was whether the exhibition of physical movements in the host species that are preferred by these mosquito vectors are less effective at deterring biting than those made by other types that they rarely select. Confirmation of this prediction would support the hypothesis that host defensive behaviours may have influenced the evolution of host specificity in this system. However, evidence of this phenomenon was mixed. *Anopheles arabiensis* had a higher feeding success on its naturally preferred cattle hosts under both feeding conditions. Furthermore this host type was the only one whose movements were not associated with a reduction in *An. arabiensis* feeding success. This suggests that either cattle have poorer behavioural responses than the other host species assayed here [19], or that *An. arabiensis* has evolved strategies to more effectively evade the behavioural responses of this host type [36]. In contrast, the feeding success of *An. gambiae s.s* was unaffected by whether hosts were free to move or behaviourally restricted for both their naturally preferred human hosts, and some rarely exploited host types (e.g. dogs, goats and calves). In both vector species, the restriction of behaviour in chickens generated the greatest proportionate increase in mosquito feeding success. This finding matches results from other mosquito systems which indicate avian hosts have considerably more effective anti-mosquito behaviours than mammals, and may explain why this host type is rarely exploited by these vectors in nature [37]. Consequently, variation in host physical behaviours may be generating selection on these malaria vectors to avoid certain host species (e.g. chickens), but cannot consistently account for their previously-documented host species preferences on the basis of experiments conducted here.

Contrary to prediction, mosquitoes feeding on hosts that were free to move during exposure either obtained larger (in the case of *An. arabiensis* and some host types for *An. gambiae* s.s.) or similarly sized blood meals than when allowed to feed from immobilized hosts. These results contrast with previous studies of the mosquito *Aedes aegypti* and other ectoparasites that have shown host physical movements reduce their blood meal size [12,21-23]. Discrepancies between these and the current study may reflect genuine biological differences in the impact of host defensive behaviour on different haematophagous insects. Another possibility is that this discrepancy is due to differences in the timing and duration of the mosquito exposure period under conditions where they were free or behaviourally limited used here. Pilot investigations in this system indicated that after landing on a host, mosquitoes require less than six minutes to initiate feeding and complete a blood meal (Lyimo *et al.* personal communication). Consequently, the 15 minute exposure period used in trials where mosquitoes were directly applied to host skin was deemed sufficient to allow mosquitoes to commence biting and feed to repletion. However, in semi-field experiments mosquitoes were exposed to freely moving hosts for a period of 12 hours (7 pm-7 am) in accord with the duration of their natural host seeking period. The enhanced feeding success of *An. arabiensis* on unrestrained hosts could be a by-product of an increased, innate predisposition for feeding during the night time hours when semi-field experiments were conducted (host restraint experiments conducted during the day). Several of the host types assayed here including humans are also more likely to be sleeping during night time hours which may have minimized the impact of any anti-mosquito defensive behaviours they are capable of making. This phenomenon could explain a lack of difference between mosquito feeding success on free and behaviourally-restrained hosts, but not the increased blood meal size of *An. arabiensis* reported here. An additional explanation for these results could be that *An. arabiensis* exposed to freely moving hosts in semi-field conditions could feed repeatedly from hosts throughout the night to top up their blood intake beyond what could be acquired from the one contact permitted in trials with immobilized hosts.

It is also possible that when mosquitoes are given the opportunity to choose where on the host body they bite (as under the semi-field, but not host behavioural restriction conditions used here), they preferentially select sites from which blood can be more efficiently imbibed. This could include areas of skin that are relatively thinner and/or blood vessels more easily accessible [38] than the locations where mosquitoes were applied under host behavioural limitation conditions. For example in experiments where host behaviour was limited, mosquitoes were applied to human forearms whereas under natural conditions they preferentially bite feet [39]. Similarly there is some evidence that *An. arabiensis* preferentially land and feed on cow legs [36], whereas here they were exposed to a variety of sites on the cow body (e.g. flanks, and thigh muscles). However, previous studies have shown that the relative ‘attractiveness’ of particular biting sites on the bodies of hosts is highly dependent on their position (e.g. *An. gambiae* s.s. preferentially bite human feet when people are sitting down, but this preference is not evident when people are lying down or have their feet in the air [39]). This suggests that there may be no intrinsically ‘optimal’ biting sites on the body of hosts, and that the variation in mosquito feeding success observed here may not be explained by treatment-specific differences in where on the host body mosquitoes were allowed to feed from. Further experiments are required to test whether the enhanced blood meal sizes associated with foraging on freely moving rather than behaviourally restricted hosts as reported here could be explained by any of these additional factors. Finally, it is noted that although the semi-field conditions used here did permit relatively realistic interactions between mosquitoes and the host types they typically encounter, they may not be fully representative of natural field conditions in which these interactions take place against a more complex background of variation in environmental conditions, host and mosquito abundance and diversity. Direct field evaluations of these hypotheses are currently problematic given their requirement to allow (potentially-infectious) mosquitoes to feed on human subjects. However if risk-free methodologies for human exposure develop, further investigation of this phenomenon under field conditions are warranted to validate results presented here.

Bearing in mind the caveats to interpretation as discussed above, the results of this study suggest that physical defensive behaviours exhibited by common host species including humans do not impose substantial fitness costs on African malaria vectors. If this is the case, alternative explanations for the evolution of host specificity in this system are needed. One possibility could be the existence of haematological variation between host species in the key traits likely to influence blood resource quality to mosquitoes (e.g. haemoglobin levels and red cell density, [40,41]. Several haematological properties such as haemoglobin concentration, red blood cell density and amino acid composition are known to be vary within and between vertebrate species [42,43], and could account for some of the variation in the fitness of haematophagous insects [9]. Laboratory experiments in which *An. gambiae* s.s. were fed blood from different host species under standardized membrane feeding conditions suggest that haematological factors alone, in the absence of additional host behavioural, ecological or physiological factors, can generate some variation in mosquito fitness [44]. However *in* the host-specific variation in mosquito fitness observed under these laboratory conditions was only partially consistent with results obtained from the more natural semi-field conditions here, and similarly did not indicate that mosquito fitness was consistently highest on the blood of preferred species. Further investigations are ongoing to evaluate the role of naturally-occurring haematological variation on the fitness of malaria vectors under more natural semi-field condition and will help resolve this issue. Other than host defensiveness and/or haematological properties, other potential explanations for the evolution of host specialization in these malaria vectors include larger-scale properties of host ecology. For example, it could be specific properties of the preferred habitats of different host types (e.g. climatic suitability of human relative to animal dwellings) that drive selection towards anthrophily, rather than innate host biological properties. Other factors such as the relative abundance and aggregation of hosts across the landscape may also be responsible for generating selection towards the types that mosquitoes most frequently encounter. Further investigation into these and other potential hypotheses are encouraged to help resolve the nature of selection acting upon malaria vector-host interactions.

As mosquito blood meal size is strongly and positively correlated with their reproductive success [45-47] and long-term survival [48], host behaviours that limit blood intake are expected to reduce these fitness traits [13]. However, the relatively larger blood meals that mosquitoes acquired from feeding on freely-moving hosts under semi-field conditions did not translate into correspondingly greater reproductive success. This may be because regardless of host species or behavioural manifestation, mosquitoes that succeeded in feeding always obtained the minimum volume of blood required to initiate oviposition [49] and maximize egg production. Anopheline fecundity is known to be linearly related to blood meal volume only above a minimum threshold below which no eggs are produced, and below a maximum threshold above which no further eggs are produced [47]. Although the larger mosquito blood meals obtained by mosquitoes under semi-field conditions were not associated with greater reproductive success, they were correlated with enhanced mosquito longevity. Blood resources are thought to be used primarily for mosquito reproduction [50-52], however some studies indicate the longevity of mosquitoes and other ectoparasites increase with ingested blood meal size [21,23,43]. Observation of a similar phenomenon here suggests these mosquito vectors also use blood proteins to synthesize energy reserves for survival [50]. Finally, it is noted that the longevity effects that were measured here only represent post-feeding survival, and not direct mortality associated with host-seeking [13]. Variation in the recapture rate of mosquitoes in semi-field trials could provide an indirect estimate of host-seeking associated mortality. However, as these recapture rates were generally similar across trials with different host species, it suggests there may be no significant variation in host-seeking mortality between host species. Previous work under the semi-field conditions suggest that mortality associated with host-seeking by these vectors is negligible [29], but further work is required to confirm this under natural field conditions.

The current up-scaling of long-lasting insecticide treated nets (LLIN) [53] and indoor residual spraying (IRS) [54] throughout sub-Saharan Africa, improvements in housing [55], and use of other protecting measures such as repellents [56] means that the relative ‘defensiveness’ of humans to malaria vectors relative to other available host types is substantially increasing. This increased protection of humans is clearly providing immediate epidemiological benefits by reducing malaria transmission [57], but may also provide longer-term ‘evolutionary’ advantages by generating selection for mosquito vectors to switch their host choice to less well defended host species [44]. Such changes are most likely to occur when mosquitoes would receive a clear fitness advantage from shifting away from humans; a process that could be undermined by the existence of strong defensive behaviours in other potential host species such as domestic animals and livestock. With the exception of chickens, no evidence was found here to suggest that the physical movements of the other animal species most likely to be kept in and around households are more costly to malaria vectors than those of humans. In fact it appears that *An. arabiensis* may encounter substantially less effective defensive behaviours when foraging on cattle than humans, and thus may do better to switch to the former host type especially if humans are universally covered with bed nets [29]. It is thus hypothesized that variation in host physical defensive behaviours are unlikely to prevent malaria vectors from exploiting alternative host species (e.g. cows) when humans are unavailable.

## Competing interests

The authors declare that they have no competing interests.

## Authors’ contributions

INL and HMF designed the research. INL, AAD, KFM and EMM performed the research. INL and HMF analyzed and interpreted the data. DTH and RR assisted with analysis. INL drafted the manuscript. DTH, RR and HMF commented on the manuscript. All authors read and approved the final manuscript.
